# Genomic and metabolic instability during long-term fermentation of an industrial *Saccharomyces cerevisiae* strain engineered for C5 sugar utilization

**DOI:** 10.3389/fbioe.2024.1357671

**Published:** 2024-03-26

**Authors:** Maëlle Duperray, Mathéo Delvenne, Jean Marie François, Frank Delvigne, Jean-Pascal Capp

**Affiliations:** ^1^ Toulouse Biotechnology Institute, INSA/University of Toulouse, CNRS, INRAE, Toulouse, France; ^2^ TERRA Research and Teaching Centre, Microbial Processes and Interactions (MiPI), Gembloux Agro-Bio Tech, University of Liège, Gembloux, Belgium; ^3^ Toulouse White Biotechnology, INSA, INRAE, CNRS, Toulouse, France

**Keywords:** phenotypic heterogeneity, genetic stability, industrial yeast strain, homologous recombination, xylose, arabinose, ethanol red, metabolic instability

## Abstract

The genetic stability and metabolic robustness of production strains is one of the key criteria for the production of bio-based products by microbial fermentation on an industrial scale. These criteria were here explored in an industrial ethanol-producer strain of *Saccharomyces cerevisiae* able to co-ferment D-xylose and L-arabinose with glucose through the chromosomal integration of several copies of pivotal genes for the use of these pentose (C5) sugars. Using batch sequential cultures in a controlled bioreactor that mimics long-term fermentation in an industrial setting, this strain was found to exhibit significant fluctuations in D-xylose and L-arabinose consumption as early as the 50th generation and beyond. These fluctuations seem not related to the few low-consumption C5 sugar clones that appeared throughout the sequential batch cultures at a frequency lower than 1.5% and that were due to the reduction in the number of copies of transgenes coding for C5 sugar assimilation enzymes. Also, subpopulations enriched with low or high *RAD52* expression, whose expression level was reported to be proportional to homologous recombination rate did not exhibit defect in C5-sugar assimilation, arguing that other mechanisms may be responsible for copy number variation of transgenes. Overall, this work highlighted the existence of genetic and metabolic instabilities in an industrial yeast which, although modest in our conditions, could be more deleterious in harsher industrial conditions, leading to reduced production performance.

## 1 Introduction

The genetic and metabolic stability of microorganisms metabolically engineered for the production of bio-based products from renewable feedstock is a critical issue in modern biotechnology (Olsson et al., 2022). It is commonly accepted that the metabolic burden caused by the expression of the heterologous or synthetic pathway, and the potential toxicity associated with the accumulation of intermediates and end products, are the main factors triggering spontaneous appearance of genotypic and/or phenotypic variants (Rugbjerg et al., 2018; Rugbjerg and Sommer, 2019). However, the mechanism underlying producer strain instability is far to be understood, as its origin can be genetic and non-genetic, with an interplay between them that complicates the study and control of strain instability (Capp, 2021). Strains engineered with artificial pathways or genetic circuits tend to lose the production phenotype in long-term fermentations due to genetic instability, metabolic burden, enzyme/cofactor imbalance, and toxic chemical accumulation (Mu and Zhang, 2023).

Previous works focused mainly on genetic stability in bacteria, where the increased frequency of mobile elements insertions in transgenes on plasmids (Heieck et al., 2023) or integrated into chromosomes (Rugbjerg et al., 2018) was shown to be the main factor in altering their capacity of production. Consequently, selective deletion of mobile elements and their corresponding transposases could prove useful for stabilizing industrially production bacterial strains (Heieck et al., 2023). In *S. cerevisiae*, homologous recombination (HR) has been recently reported as an important genetic process leading to the loss of production capacity (Lee et al., 2021; De Mol et al., 2023), as illustrated in the case of vanillin-β-glucoside (D'Ambrosio et al., 2020). In most cases, this HR-induced instability of producer strains is due to the easy excision of heterologous genes encoding enzymes of the pathway that are integrated into the genome in multicopy with identical promoter/terminator (Rugbjerg and Sommer, 2019). In addition to genetic instability, the last decade has shed light on non-genetic sources of strain instability, which have been attributed to cell-to-cell heterogeneity. Notably, metabolic heterogeneity with clonal microbial cells varying in metabolite levels (Xiao et al., 2016) and metabolic fluxes has been highlighted (Delvigne et al., 2014). For instance, metabolic instability was revealed in a naringenin-producing yeast engineered by multiple gene integrations in which naringenin production declined rapidly after 200 generations (Lv et al., 2020). A strategy consisting in maintaining a selective growth advantage to enrich the producing populations through elimination of cells that produce less naringenin maintained 90.9% of naringenin titer up to 324 generations. Causal factors range from intracellular molecular noise including stochastic gene expression (Takhaveev and Heinemann, 2018) to environmental factors (Schreiber and Ackermann, 2020). In particular, noise in the expression of genes involved in DNA repair and recombination (Capp, 2010) can lead to cell-to-cell heterogeneity in both bacteria and yeast (Uphoff et al., 2016; Liu et al., 2019). For instance, we recently reported that spontaneous HR rates could vary up to 10-fold in a *Saccharomyces cerevisiae* clonal population depending on the level of expression of genes affecting HR activity either directly (such as *RAD52* involved in the early steps of HR pathways) or indirectly (such as *RAD27* involved in Okasaki fragments maturation) (Liu et al., 2019). This HR-dependent cell-to-cell heterogeneity can therefore favor the emergence of adaptive behavior under extreme conditions, as well as trigger the appearance of poorly performing variants.

In this report, we set out to explore the genomic and metabolic stability of an industrial ethanol-producing strain engineered to co-ferment C5 sugars (xylose and arabinose) with glucose in long-term culture. We found that the rate of xylose and arabinose consumption started to fluctuate after around 50 generations without any major genomic modifications detected at the population level. In addition, clones that exhibited reduced or complete loss of arabinose and/or xylose assimilation were isolated from the bulk population. Our results indicated that the phenotype of these variants was due to variation in the copy number of transgenes encoding enzymes in the C5-sugar assimilation pathway. Even though these variants represented less than 1.5% of the total population, this event may lead to the loss of the fermentative capacity of these sugars in a longer term and under industrial conditions that are harsher than those experienced in this study.

## 2 Material and methods

### 2.1 Strains, plasmids and media

All experiments were performed using the *S. cerevisiae* HDY.GUF12 strain (Dietz, 2013; Generoso et al., 2017) (Lesaffre France). The HDY.GUF12 strain containing the *RAD52-YFP-tdTomato* fusion was obtained using a CRISPR-Cas9 strategy (Ryan et al., 2016). The high copy pCas9-amdSYM plasmid [derived from pML107 (Laughery et al., 2015)], which constitutively expresses the gene encoding the Cas9 endonuclease and carrying a guide RNA (gRNA) expression cassette, was used to transform the HDY.GUF12 strain. A healing fragment containing *YFP-tdTomato-SpHis5* and homologies to *RAD52* was amplified from the JA0241 strain (Liu et al., 2019) (primers GAT​CCC​AAA​TAC​CAG​GCA​CA and CAT​AGT​TCA​ATT​GCG​TGA​CAT​C) and used to repair the double-strand break introduced by the Cas9 endonuclease. The target sequence of the gRNA was identified using the online software CRISPR-direct (https://crispr.dbcls.jp), and located in the *RAD52* 3′UTR region. Ligation of the gRNA expression cassette sequence into the pCas9-amdSYM plasmid was made using a T4 DNA ligase (NEB). Yeast cells were transformed using the procedure of (Dietz, 2013). Transformants were selected on YNB Acetamide plates (1.7 g/L yeast nitrogen base without amino acids and nitrogen (Euromedex), 6.6 g/L K_2_SO_4_, 0.6 g/L acetamide and 20 g/L D-glucose) as the amdSYM cassette confers the ability to use acetamide as the sole nitrogen source (Solis-Escalante et al., 2013). Strain and plasmid constructions were verified by PCR amplification and sequencing. A synthetic mineral yeast medium adapted from the Verduyn recipe described in (Verduyn et al., 1992) was used for the serial cultivations in microplates. This medium, named SHD2, contained 5-times more trace elements than the original recipe, 0.1 M potassium hydrogen phthalate, 16 g/L D-glucose, 13 g/L D-xylose and 11 g/L L-arabinose and adjusted at pH 5.0. YPD medium contained 20 g/L D-glucose, 20 g/L bactopeptone and 10 g/L yeast extract. YNB medium contained 1.7 g/L yeast nitrogen base without amino acids and nitrogen (Euromedex), 5 g/L ammonium sulphate and was supplemented with appropriate sugars, as indicated in the following sections.

### 2.2 Batch fermentation

Cultivations were performed in 750 mL 2X YNB medium supplemented with 60 g/L D-glucose, 26 g/L D-xylose and 22 g/L L-arabinose in a 1 L bioreactor (Solaris Biotech Solutions). Agitation and temperature were set at 400 rpm and 30°C respectively and pH was maintained at 5.5. Airflow was maintained at 100 mL/min during the first 18 h (until all glucose was consumed), before assigned anaerobic conditions (N2 injection). Cells were inoculated in the first batch at OD = 0.1 from overnight pre-culture of the WT HDY.GUF12 strain and cultivated for 48 h before being inoculated in the next batch at the same initial OD. Serial batch cultivations were performed in duplicate allowing to reach 90 (15 successive batch) or 96 (16 successive batch) (Supplementary Table S1). Samples were regularly collected all along cultivations to perform OD measurements and further metabolite quantifications. At the end of each batch, cells were also stocked in glycerol solution and kept at −80°C.

### 2.3 Successive cultivation in microplates and cell sorting

To initiate the successive cultures in microplates, cell suspensions of each strain (WT and *RAD52-YFP-tdTomato* strains) obtained from overnight pre-cultures in SHD2 medium, were inoculated in a 48-well FlowerPlate (800 μL at OD600 = 0.1). Cultures were performed in the Biolector Pro (Beckman Coulter GmbH, Germany) at 30°C in SHD2 medium, under propagation conditions for 24 h at a shaking frequency of 1,000 min^−1^ to ensure a high oxygen transfer rate. This was followed by cultivation under production conditions for the last 48 h at a shaking frequency of 800 min^−1^ after the addition of 800 µL of fresh medium to decrease the oxygen transfer rate. After this initial culture cycle (C0), OD (600 nm) was measured, cells were harvested and the supernatant was kept at −20°C for further HPLC analysis while the pellet was resuspended in PBS and kept at −20°C before cell sorting. At this stage, cells were also stocked in glycerol solution and kept at −80°C. The cells were sorted using a FACS Aria III (BD biosciences, Belgium) based on a custom mode (0–16–0) with an 85 µm nozzle. A first gate was applied to select cells based on YFP (488 nm laser, 530/30 filter) and tdTomato (561 nm laser, 582/15 filter) fluorescence signals. Based on the FSC-Area vs. FSC-Height (561 nm laser) plot, single cells with similar cell size and granularity were then selected. Finally, based on the histogram of the YFP-tdTomato fluorescence (561 nm laser, 582/15 filter), single cells with the 10% highest (renamed Rad52-high) and the 10% lowest (renamed Rad52-low) Rad52-YFP-tdTomato fluorescence levels were sorted simultaneously and recovered in PBS. The WT strain was sorted without this final gating (100% WT single cells). After cell sorting, recovered cells [approximately 2.10^5^ cells for each strain (Supplementary Table S2 for details)] were harvested (10,000 g during 30 min) and resuspended in 800 µL of fresh medium to be inoculated in a 48-well FlowerPlate. These cells were cultivated, sampled and sorted as the C0 culture cycle, except in the following steps: propagation condition lasted 48 h; only the high-Rad52 cells were recovered from the cultures of Rad52-high, while only the low-Rad52 cells were recovered from the cultures of Rad52-low, thus enriching the cultures with high- or low-Rad52 cells respectively. A total of nine culture cycles have been performed for each condition (Rad52-low, Rad52-high and WT strain) to reach more than 90 generations.

### 2.4 Replica tests and growth analysis by spot assays

About 1,500 cells from an overnight culture were spread on YNB plates containing 20 g/L D-glucose, 10 g/L D-xylose and 10 g/L L-arabinose. The clones from each plate were then transferred by replica plating on YNB plates containing 20 g/L D-glucose, 20 g/L D-xylose or 20 g/L L-arabinose. Clones exhibiting a growth difference on xylose and/or arabinose medium to that on glucose medium were selected and further analyzed by spot assays. They were grown overnight on YPD plates and resuspended in sterile MilliQ water at 2.10^7^ cells/mL before being submitted to 1/10 serial dilutions. Drops (5 *μ*L) of each dilution were spotted onto freshly prepared YNB plates containing 20 g/L D-glucose, 20 g/L D-xylose or 20 g/L L-arabinose and were incubated at 30°C.

### 2.5 Analytical methods for metabolites

Supernatants from cultures were syringe filtered (0.2 µm) before analysis using HPLC. Samples from the successive cultivations in microplate were analyzed using an HPLC Agilent 1,200 Series (Agilent) equipped with an Aminex HPX-87H column coupled with a pre-column (Bio-Rad). The other samples were analyzed using either Vanquish or Ultimate 3000 HPLC system (Thermo Scientific) equipped with a REZEX ROA-Organic Acid H^+^ column coupled with a pre-column (Phenomenex). For all experiments, the eluent solution was 5 mM H_2_SO_4_, running at 0.5 mL/min for 40 min at 50°C. Compounds were detected by an RI detector and quantified from standard curves using the Chromeleon software.

### 2.6 Real-time PCR assays

Genomic DNA was extracted using the MasterPure Yeast DNA Purification kit (LGC Biosearch Technologies) and quantified by NanoDrop (Thermo Scientific). The copy number of the five genes of interest was determined using the MyIQ real-time PCR system from Bio-Rad. The reaction mix (25 *μ*L final volume) contained 12.5 *μ*L of iQ SYBR Green Supermix buffer (Bio-Rad), 3 *μ*L of each primer (Supplementary Table S3) at a final concentration of 250 nM and 10 ng of DNA. The thermocycling program consisted of one hold at 95°C for 5 min, followed by 40 cycles of 10 s at 95 °C and 45 s at 56°C. To correlate the copy number of each gene to the Ct value, calibration curves were performed with each couple of primers on serial dilutions of DNA from the WT strain (Supplementary Figure S5). Genome copy number was first determined using the *ACT1* gene as a reference, allowing us to calculate the copy number of each gene of interest. The resulting data were then normalized to the WT initial strain condition for which copy number of genes is known.

### 2.7 Genome sequencing

Cells were cultivated in YPD medium directly from glycerol stock to obtain 10^9^ cells. Genomic DNA was extracted using the Blood and Cell Culture DNA Midi Kit (Qiagen) according the manufacturer’s protocol adapted for yeast cells with following modifications: (i) lyticase digestion extended to 2 h; (ii) proteinase K digestion extended to 3 h; (iii) cellular debris pelleted by centrifugation at 5,000 *g* for 20 min with supernatant filtered (0.2 µm) before applying to the equilibrated genomic-tip; (iv) DNA precipitated in isopropanol was pelleted by centrifugation at 5,000 g for 20 min, washed two-times in cold ethanol 70%, dried at 37°C for 30 min and dissolved overnight in EB buffer (Qiagen). DNA was quantified by NanoDrop (Thermo Scientific) and its quality control was validated on Qubit (Thermo Scientific) and Fragment Analyzer (Agilent).

Single-molecule real-time long-reads sequencing was performed at Gentyane Sequencing Platform (Clermont-Ferrand, France) with a PacBio Sequel II Sequencer (Pacific Biosciences, Menlo Park, CA, United States of America). The SMRTBell library was prepared using an SMRTbell prep kit 3.0, following the procedure and checklist -preparing whole genome and metagenome libraries protocol. Genomic DNA (1 µg) of each strain was sheared using g-tubes (Covaris, England) generating DNA fragments of approximately 10 kb. Sheared genomic DNA was carried into the enzymatic reactions to remove the single-strand overhangs and to repair any damage that may be present on the DNA backbone. An A-tailing reaction followed by the barcoded overhang adapter ligation was conducted to generate the SMRT Bell templates. After nuclease treatment, the sample was then size-selected for fragments above 5 kb with 35% AMPure PB Beads and equimolar multiplied to obtain the final libraries around 10 kb. A ready-to-sequence SMRTBell Polymerase Complex was created using a Binding Kit 3.2 (PacBio) and the Sequel II primer 3.2. The PacBio Sequel instrument was programmed to load a 90 p.m. library and sequenced in CCS mode on a PacBio SMRTcell 8M, with the Sequencing Plate 2.0 (Pacific Biosciences), 2 h of pre-extension time and acquiring one movie of 15 h per SMRTcell.

### 2.8 Bioinformatics analysis

The CCS tool (v6.3.0) from the smrttools suite (11.0.0.146107), with default parameters, was used to generate the corrected CCS data (20.03 Gb, mean 8,880 bp) from raw data. These CCS were demultiplexed per sample using lima (2.5.1). Assembly with hifiasm (0.16.1-r375) was performed to obtain the two haplotypes of diploid strains. Each contig was aligned against the reference strain S288C, using nucmer (3.1). Thus, a chromosome name could be assigned to each contig, based on the percentage identity between S288C and the hifiasm assemblies. The pbmm2 tool (2.8.1) was used to align the pools of reads against the diploid reference assembly (*RAD52-YFP-tdTomato* strain). The pbsv pipeline (2.8.1) was used to call structural variants with a minimal size set at two nucleotides. The various vcf calling files were collected and analysed using VCFtools (0.1.16). The various SVs found were visualised and confirmed using IGV.

## 3 Results and discussion

### 3.1 Identification of C5-sugar assimilation instability during long-term fermentation of an industrial strain

**FIGURE 1:**
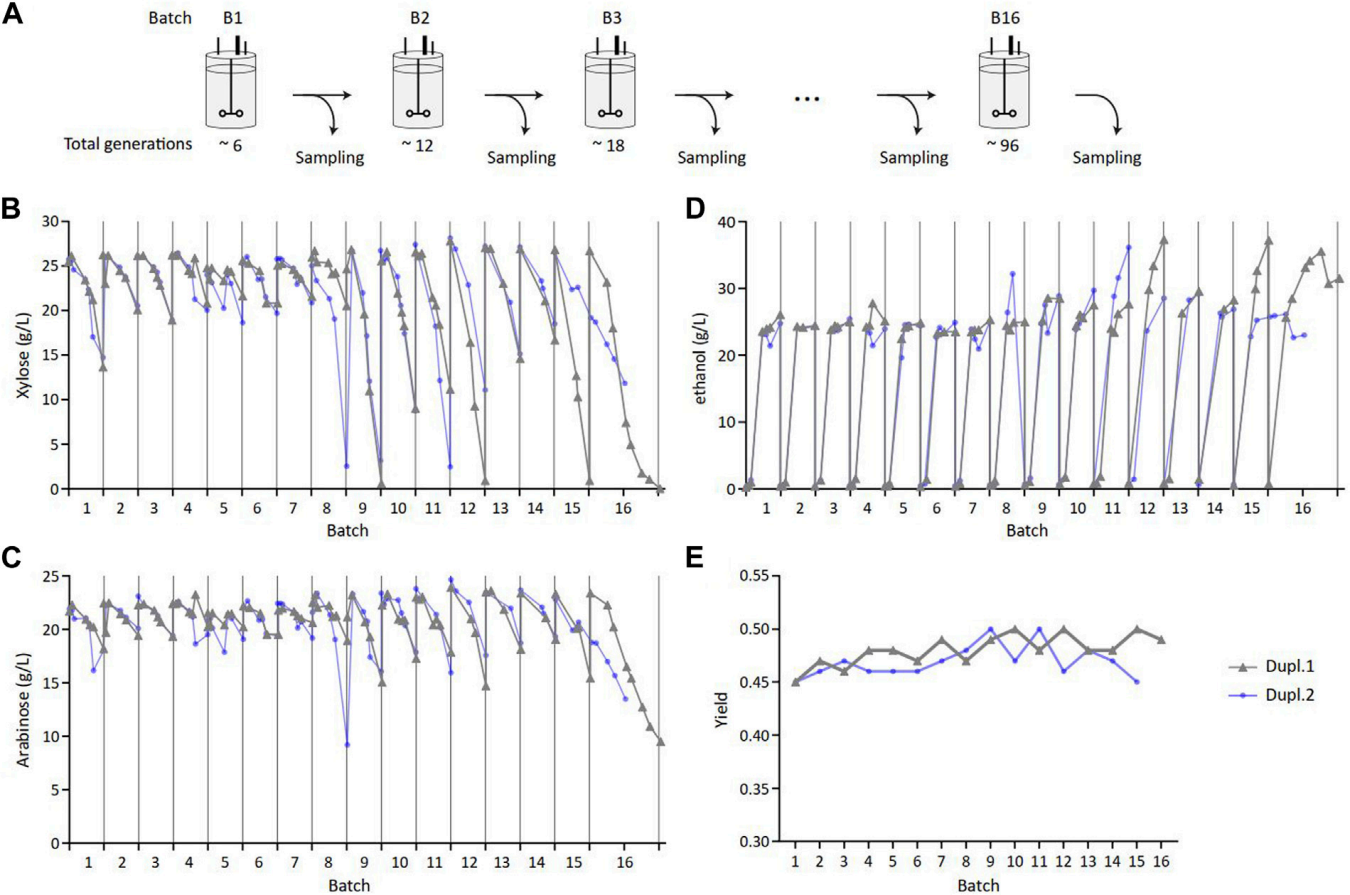
Heterogeneity in xylose and arabinose consumption and ethanol production in sequential batch cultures set up. **(A)** Serial cultivations performed in batch reactor to reach 96 generations. Samples from two parallel cultivations (Dupl.1 and Dupl.2) were regularly collected for subsequent analyses. Extracellular xylose **(B)**, arabinose **(C)** and ethanol **(D)** quantification along cultivations. **(E)** Ethanol yield estimated from the last measures of each batch and calculated by dividing the total number of carbon produced by the total number of carbon consumed from glucose, xylose and arabinose.

Long-term sugar fermentation into ethanol was carried out using the industrial ethanol-producing HDY.GUF12 strain. This strain derived from the diploid ethanol Red^®^ strain (Jacobus et al., 2021) that was previously metabolically engineered to ferment D-xylose and L-arabinose by several chromosomal insertions of the genes encoding enzymes of the C5-sugar assimilation (Supplementary Figure S1) and evolutionary engineering to optimize the co-fermentation of these sugars together with glucose (Dietz, 2013; Demeke, 2013). This strain was grown in duplicate (Dupl.1 and Dupl.2) in 16 successive batch bioreactors that corresponded to about 100 generations (Figure 1A; Supplementary Table S1), which approximately matched the number of generations from the starting inoculum to the industrial fermentation process (Rugbjerg and Sommer, 2019). Each batch took 48 h with an aerobic phase that lasted about 18 h during which glucose was totally consumed (not shown), followed by an anaerobic phase during which xylose and arabinose were fermented into ethanol. As shown in Figures 1B, C, the consumption of these C5 sugars fluctuated between batches, starting at the 50th generation and ranging from low, to total consumption. The same trend was observed for ethanol production (Figure 1D). In addition, the yield of ethanol was measured in the range of 45%–50% of sugars consumed all over the successive batch (Figure 1E). It was also verified that the ethanol tolerance of the cell population taken along the successive batches was comparable (Supplementary Figure S2), indicating that observed fluctuations in C5 consumption was not associated with a toxic effect due to high ethanol production but rather due to differences in the ability to consume xylose and arabinose.

This C5 consumption pattern contrasted with previous studies reporting a complete consumption of xylose and arabinose with this strain in other experimental conditions (Dietz, 2013). Nevertheless, these highly variable and unpredictable fluctuations in C5 sugar consumption are unlikely to be of genetic origin, as the duration of each batch fermentation is too short to generate genetic variants harboring this phenotypic trait. This was confirmed by whole genome long-read sequencing performed on samples coming from four batch cultures (B5, B9, B13 and B16 for Dupl.1; B5, B9, B12 and B15 for Dupl.2), which revealed no genomic differences between the tested samples at the population level. Therefore, the phenotypic fluctuations we observed could arise from epigenetic events that could modulate the expression level of the transgenes or other endogenous genes involved in C5 sugar consumption (Francois et al., 2020).

**FIGURE 2:**
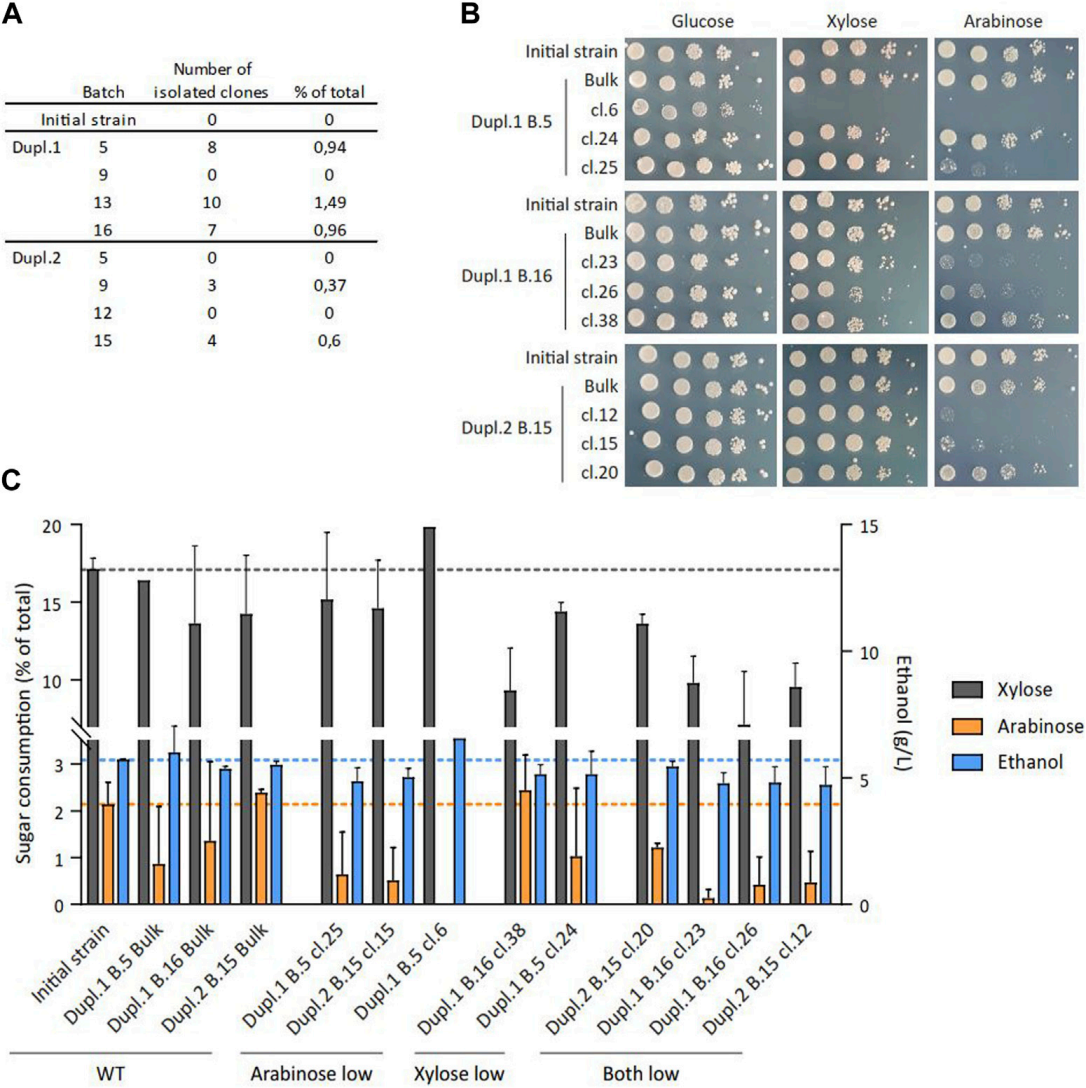
Phenotypes of clones isolated from successive batch cultures. **(A)** Summary of the number of identified clones per tested batch exhibiting a growth defect on xylose and/or arabinose. **(B)** Spotting assays of serial dilutions of cell suspension on YNB plates containing the indicated sugar at a final concentration of 20 g/L for nine selected clones. **(C)** Extracellular xylose, arabinose and ethanol quantification after 24 h on YNB medium containing 16 g/L D-glucose, 13 g/L D-xylose and 11 g/L L-arabinose for nine selected clones (n = 2).

This unexpected result on the fluctuated consumption of C5 sugars during successive fermentation batches prompted us to examine the possible existence of clones within the population with a differential capacity to metabolize these sugars. To this end, cells samples from eight different batches (B5, B9, B13 and B16 for Dupl.1; B5, B9, B12 and B15 for Dupl.2) were spread on plates containing glucose, xylose and arabinose to get about 1,000 individual clones per batch that were replica-plated on media containing only glucose, xylose or arabinose. This experiment unraveled several clones exhibiting a differential growth on xylose and/or arabinose as compared to glucose, which were further analyzed by spotting serial cell dilutions. A total of 32 clones exhibiting alteration of C5-sugar assimilation were isolated and represented up to 1.5% of the total tested clones per batch (Figure 2A; Supplementary Figure S3). Two-thirds of them were affected in their ability to grow on xylose and all of them were affected in their ability to grow on arabinose (Supplementary Figure S3). This alteration was confirmed in nine of these clones (Figure 2B) which exhibited a significant reduction of C5-sugar consumption and ethanol production after 24 h of cultivation, compared to the initial strain and the bulk population (Figure 2C). Particularly, ethanol production correlated with C5 sugar consumption (Supplementary Figure S4), suggesting the ethanol yield of these clones was unaffected whatever the C5 sugar consumption level.

### 3.2 The low C5-fermenting clones in the bulk population of the industrial strains are due to copy number variation of the transgenes

**FIGURE 3:**
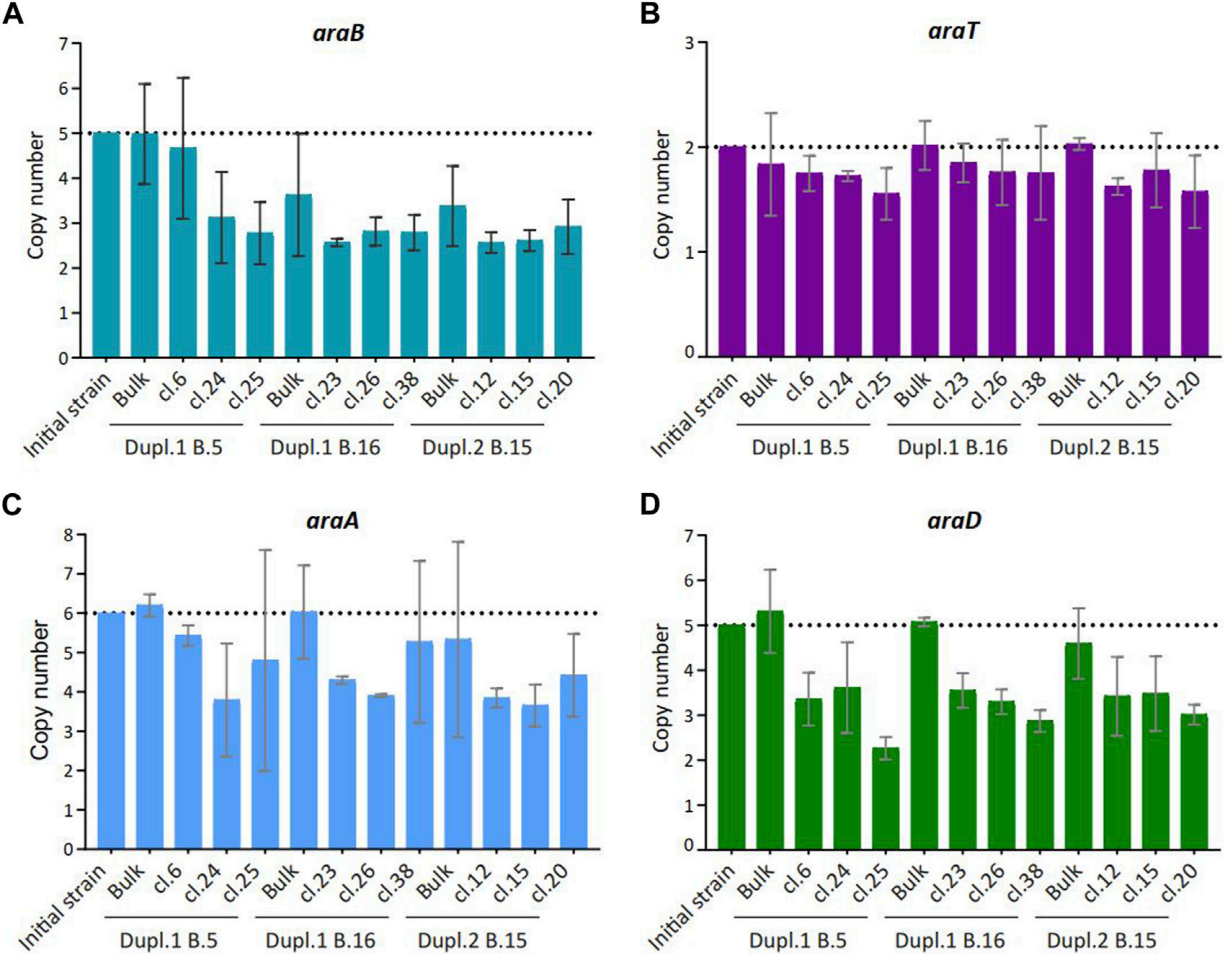
Copy number of transgenes involved in arabinose consumption in selected clones isolated from batch cultures. Copy number of *araB* **(A)**, *araT* **(B)**, *araA* **(C)** and *araD* **(D)** were determined by qPCR. *ACT1* served as a reference gene and data were normalized to the WT initial strain condition (n = 3).

We wondered whether the occurrence of low-consuming C5-sugars isolated clones in the bulk population could be a consequence of a loss of transgenes involved in their metabolization. As most of the clones retained in the study exhibited an arabinose low-consuming phenotype, RT-PCR method was used as the appropriate method to determine the copy numbers of the arabinose-associated transgenes *araA*, *araB*, *araD* and *araT*, using *ACT1* as a reference for normalization (Figure 3), because absolute quantitative PCR is currently used to quantify the gene copy number in transgenic yeast (Shirvani et al., 2020). As reported in Figure 3, the copy number of these genes was reduced, with *araB* encoding L-ribulokinase being the most affected (i.e., from 5 to two copies in most of the clones), followed by *araA,* encoding the arabinose isomerase and *araD* encoding the epimerase converting L-ribulose into xylulose, whereas the copy number of *araT* that encodes the arabinose transporter showed no variation. Previous work has shown that the rate for C5 sugar assimilation was correlated with transgene copy number (Dietz, 2013), suggesting that the decrease in the arabinose-associated transgenes copy number we observed can explain the low-consuming phenotype in the isolated clones. Thus, all along cultivation, rare cells in the population displayed genome modification, at least in transgene copy number, that affected their ability to consume C5 sugars.

Taken together, these results unraveled the existence of low-consuming C5-sugars clones that have escaped our initial genome sequencing at the population level, likely because of the low abundance of these clones that did not exceed 1.5% of the whole population. In addition, the abundance of these low-C5 sugar consuming clones did not statistically change along the successive fermentation batches, suggesting that this rare event may arise from an intrinsic genetic instability of the engineered strain. Nevertheless, it cannot be excluded that in a longer term, the abundance of these variants would increase, especially under anaerobiosis taking into account that the energetic provision from C5 sugars is 12.5% less per mole sugar than from glucose. Thus, cells losing C5-fermentation could have a fitness advantage over those keeping this ability. The existence of this putative low level of basic genetic instability could lead to genetic drift in the population, particularly in industrial bioreactors where culture conditions are more stringent.

### 3.3 Enrichment in cells expressing high Rad52 levels does not increase genomic instability

**FIGURE 4:**
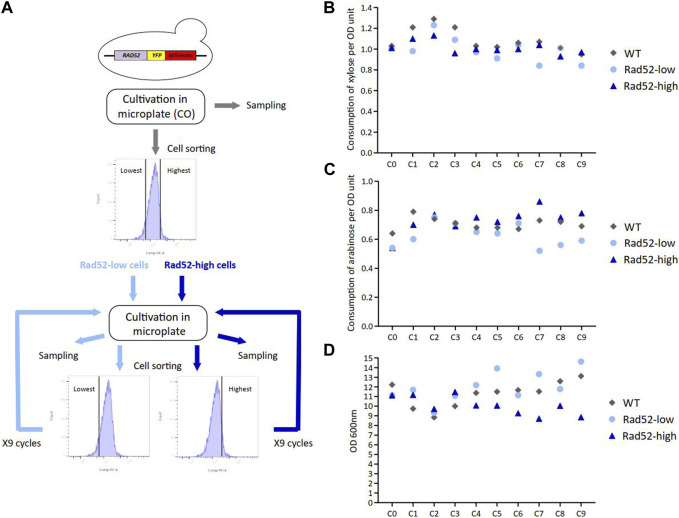
Serial cultivations of subpopulations enriched in either high- or low-Rad52 expressing cells. Experimental workflow of the successive cell sorting based on the Rad52 expression level and cultivations in microplate experiment. Samples were collected at the end of each cycle for subsequent analysis **(A)**. Extracellular xylose **(B)**, arabinose **(C)** and OD **(D)** quantification from samples collected at the end of each cycle.

In yeast, copy number variations (CNVs) are mostly due to HR events (Zhang et al., 2013). These events are expected to be more frequent when transgenes are inserted in multiple copies or where identical promoters and terminators are present within the expression cassettes such as in HDY.GUF12 (Supplementary Figure S1) because they constitute repeated sequences and could be responsible for the observed CNV (Hastings et al., 2009). It has been previously shown that the rate of spontaneous HR in *S. cerevisiae* clonal population positively correlates with the expression level of genes involved in HR activity such as *RAD52* (Liu et al., 2019). To investigate whether the subpopulations expressing more *RAD52* accumulate more genomic modifications, and thus concurrently harbored defects in C5-sugar assimilation, we performed successive cell sorting consisting of regularly enriching the population either in high- or low-Rad52 expressing cells. Cells were successively cultivated in microplates and sorted based on the expression of Rad52 thanks to the integration of the *YFP-tdTomato* reporter cassette in 5’ at the *RAD52* original locus. Nine cycles of cultivations were performed with successive sorting of the 10% Rad52-highest expressing cells (Rad52-high) and the 10% Rad52-lowest expressing cells (Rad52-low) in parallel, while the untagged strain (WT) was cultivated without cell sorting (Figure 4A; Supplementary Figure S6). These cycles of cultivations allowed us to reach more than 90 generations for each condition (Supplementary Table S2) with significant enrichment in high- and low-Rad52 cells respectively, as shown by the fluorescence profile of the populations (Supplementary Figure S7). The consumption of C5 sugars was determined at the end of each cycle, and no difference was detected between the three populations (Figures 4B, C), indicating that the enrichment in Rad52-high cells did not lead to visible phenotypic effects in terms of sugar consumption. Of note, the final OD for Rad52-high populations progressively decreased compared to the Rad52-low and the non-sorted populations (Figure 4D; Supplementary Table S2), which could be due to high expression of the *YFP-tdTomato*, high Rad52 expression levels or indirect consequences of these high gene expressions that may cause some metabolic burden.

We then wondered if this enrichment led to the appearance of genomic modifications in the Rad52-high population during cultivation despite the absence of phenotypic effects on sugar consumption. We performed long-read sequencing on samples from the C0 and C9 cycles for the WT strain and from the C0, C3, C7 and C9 cycles for the Rad52-high and Rad52-low cultures. We did not identify any genomic modification including in the transgenes in the selected Rad52-high cultures, in accordance with stability in C5 consumption. However, three losses of heterozygosity (LOH) in the WT strain from the C9 culture compared to the C0 culture were found (Supplementary Table S4). These genetic modifications probably occurred through HR and invaded the population of this strain. Moreover, we identified two LOH regarding transposon sequences in the Rad52-low population from the C3, C7, and C9 cultures. To conclude, we did not detect any enrichment of HR-linked genomic modifications in the Rad52-high cells, suggesting that other factors than heterogeneity in *RAD52* expression are responsible for the CNVs observed in the industrial WT strain. Alternatively, the cell populations were not cultivated long enough or in sufficiently challenging conditions to enable the enrichment of HR-related genomic events (Liu et al., 2019). A recent work of De Mol et al. (De Mol et al., 2023) showed that the multicopy insertion of the yeast-enhanced cyan fluorescent protein (yECFP) reporter gene into the yeast genome had a negative effect on the yECFP output. They attributed this negative effect to the variation in the number of copies of this gene and suggested that HR could play a major role in this process by generating this genetic instability. Our work did not reveal any effect of HR on the copy number of the transgenes in the producer strain, even in cells enriched with higher HR, suggesting that other factors such as epigenetic phenomena could contribute to varying extents to this decrease in output as also proposed by the same authors (De Mol et al., 2023). Interestingly, previous adaptive laboratory evolution experiments in a recombinant industrial *S. cerevisiae* strain engineered to improve xylose and arabinose co-utilization did not revealed any mutation in the heterologous genes of the pentose converting enzymes in the evolved strain compared to its parental strains (Garcia Sanchez et al., 2010). On the other hand, the observed increased activities of the xylose reductase and xylitol dehydrogenase of this pentose pathway was suggested to result from one or several duplcations on the genome of the *XYL1* and *XYL2* genes. Altogether, these results indicated CNV are a common mechanism occurring to tune the activities of these heterologous pathways.

## 4 Conclusion

This work was dedicated to explore the genetic and metabolic stability of an industrial yeast strain metabolically engineered for co-fermentation of C5-sugar with glucose under sequential fermentation batch set-up mimicking the time and generation number required for an industrial production process. Our results unravel two insights that could penalize the long-term fermentation performance of the industrial strain. Firstly, D-xylose and L-arabinose assimilation showed significant fluctuations from the 50th generation onwards, and this fluctuation, which is unlikely of genetic origin, could lead to a loss of performance over a longer term. Secondly, loss or reduction in the consumption of C5 sugar of a tiny number of cells in the bulk population has been uncovered. This phenotypic event was associated with copy number variations in transgenes involved in C5-sugar assimilation.

Although in this work we report very modest genetic and metabolic instabilities that did not affect the fermentative robustness of the yeast strain, it cannot be ruled out that these instabilities would be exacerbated under industrial production conditions and eventually lead to the loss of this robustness. Therefore, specific tools designed to evaluate these variations should be relevant in an industrial context, together with methods that enable to abolish the basal genetic instability in order to ensure long-term strain robustness and performance.

## Data Availability

The raw data supporting the conclusion of this article will be made available by the authors, without undue reservation.
