# Supplementary material for: Genomic and metabolic instability during long-term fermentation of an industrial Saccharomyces cerevisiae strain engineered for C5 sugar utilization
Source: Front Bioeng Biotechnol. 2024 Mar 26;12:1357671. doi: 10.3389/fbioe.2024.1357671 (PMC11002265; doi:10.3389/fbioe.2024.1357671)
Supplement: Supplementary file 1 [file DataSheet1.docx]

Supplementary Material

**Genomic and metabolic instability during long-term fermentation of an industrial *Saccharomyces cerevisiae* strain engineered for C5 sugar utilization.**

Maëlle Duperray, Mathéo Delvenne, Jean Marie François, Frank Delvigne and

Jean-Pascal Capp*

*** Correspondence:** [capp@insa-toulouse.fr](mailto:capp@insa-toulouse.fr)


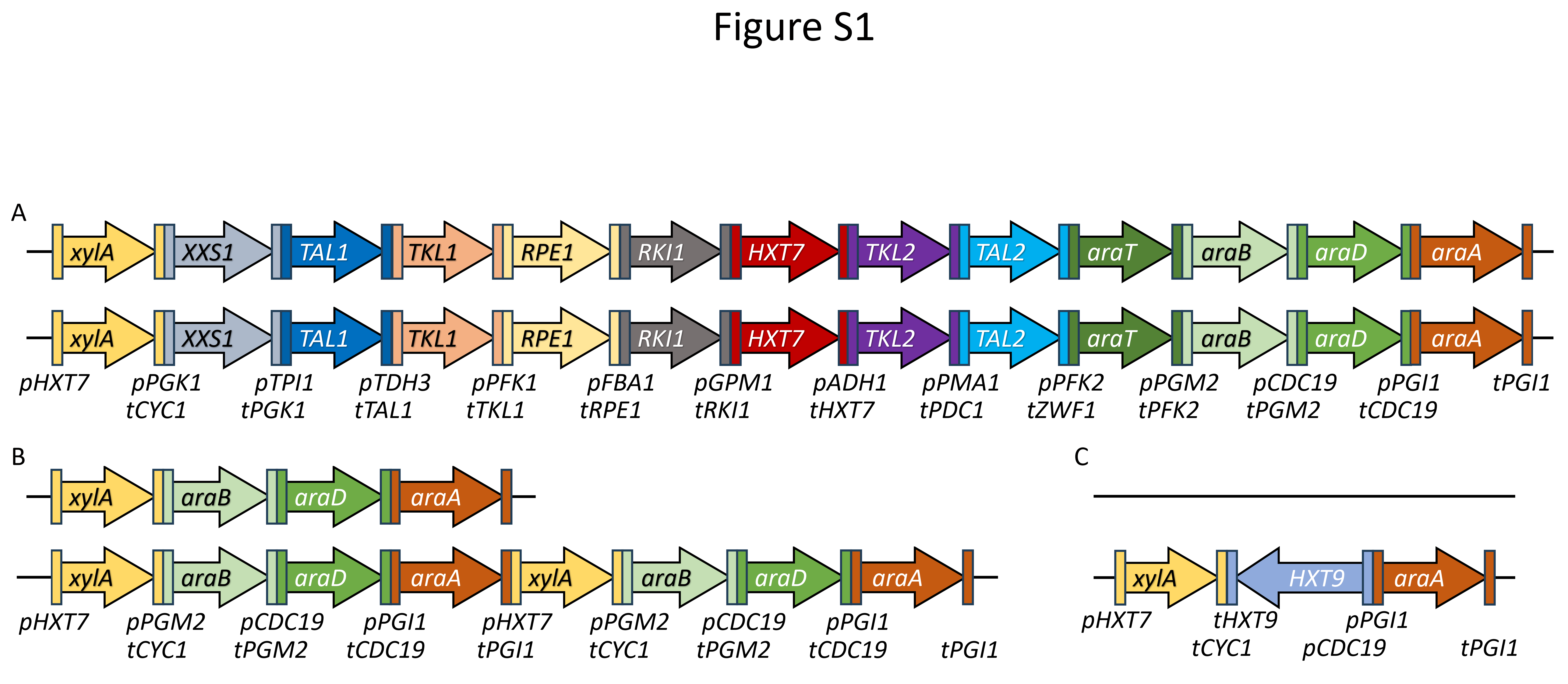


**Supplementary Figure 1.** **Genome-integrated transgenes of the HDY.GUF12 strain.**

The different cassettes introduced in the *PYK2* (A), *GAL2* (B) and *HXT2* (C) loci are represented by an arrow, each containing the ORF of the indicated gene and a distinct yeast promoter and terminator. Lines represent the native chromosome. The two distinct chromosomal copies are represented for each locus.


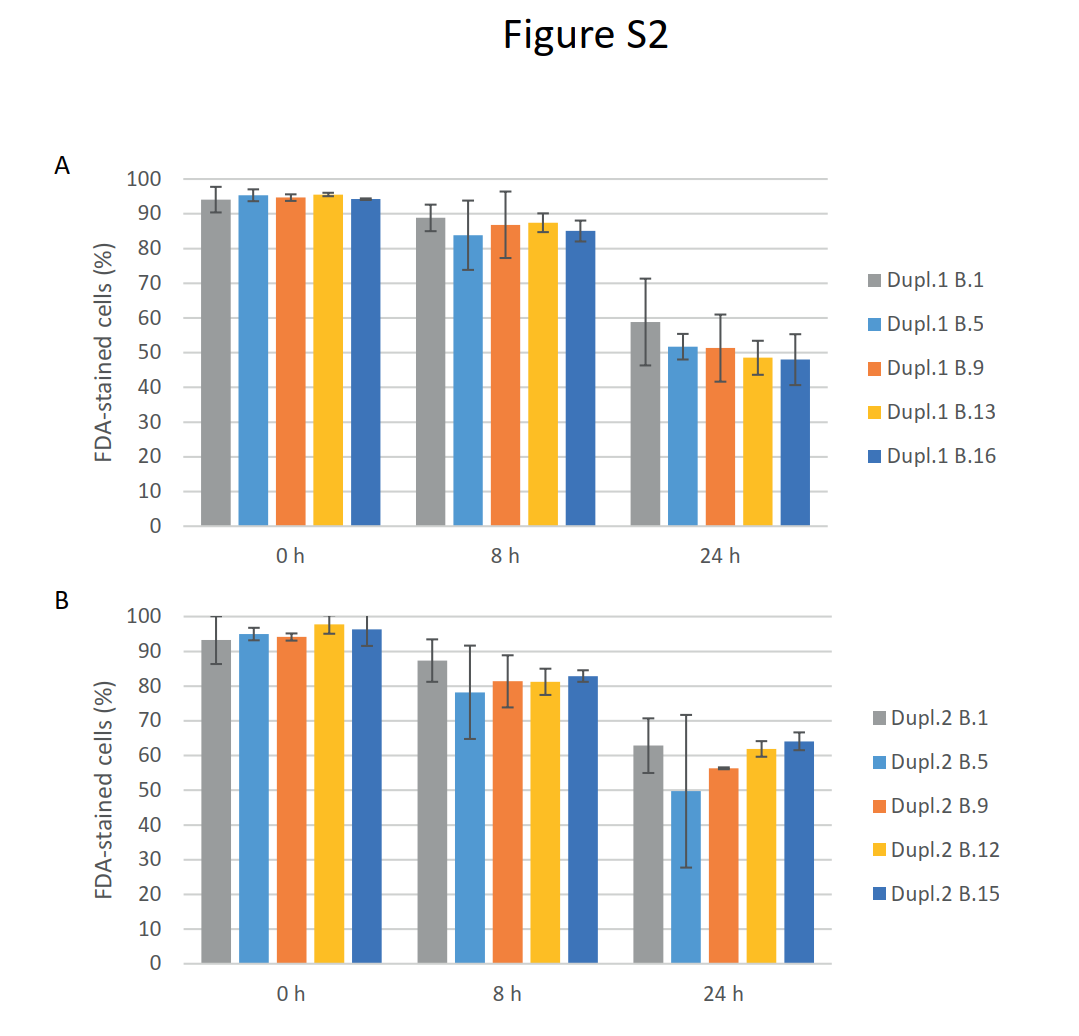


**Supplementary Figure 2.** **Ethanol tolerance of bulk population from different successive batch cultures.**

Cells from Duplicate.1 (A) or Duplicate.2 (B) were cultivated in YNB medium containing 20 g/L D-glucose, 10 g/L D-xylose and 10 g/L L-arabinose and supplemented with 31.5 g/L ethanol during 24 h. About 5.10^6^ cells were collected, washed in PBS and stained using fluorescein diacetate (FDA) at a final concentration of 20 μg/mL to assess cell vitality. The number of stained cells was determined by flow cytometry (excitation with the 488 nm blue laser and emission detection with the FL1 533/30 nm filter) using an Accuri C6 Plus cytometer (Becton–Dickinson) (n>=2).


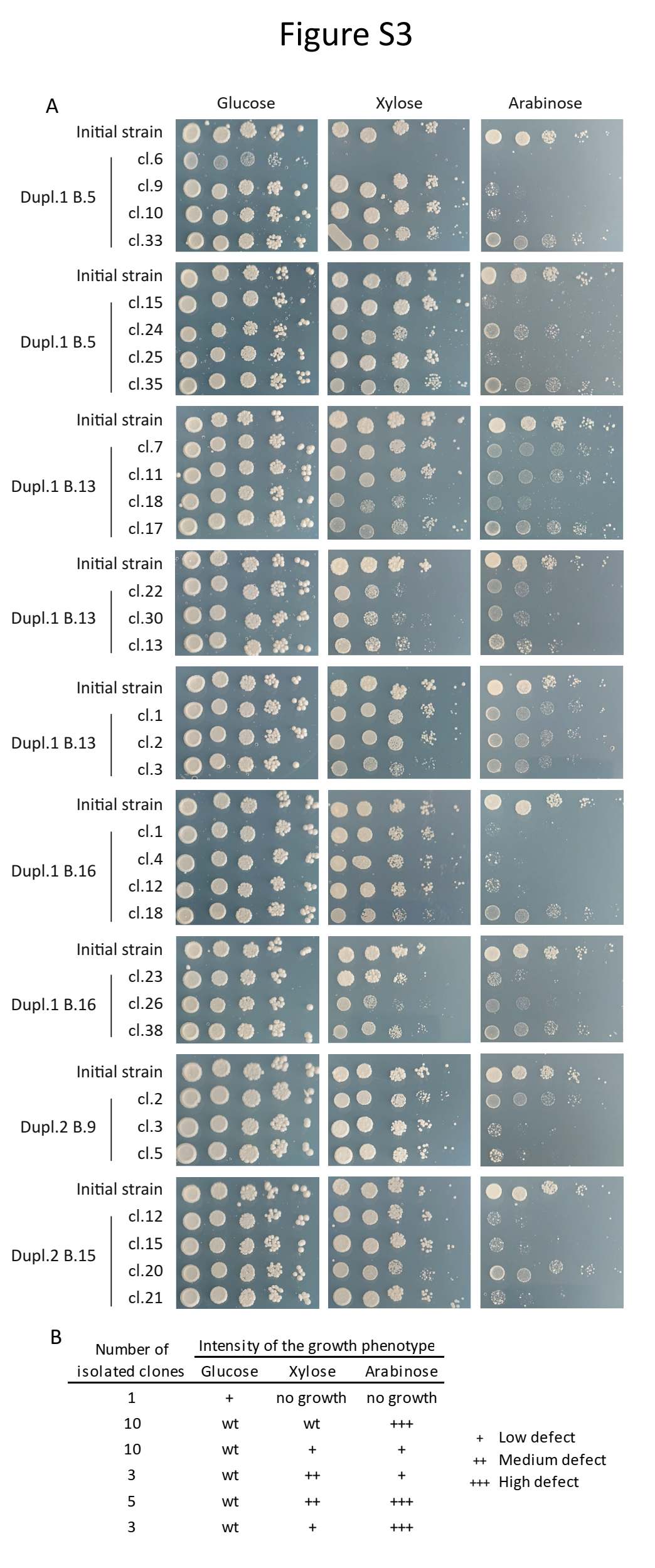


**Supplementary Figure 3.** **Growth phenotype of clones isolated from successive batch cultures.**

A: Spotting assays of serial dilutions of cell suspension on YNB plates containing the indicated sugar at a final concentration of 20g/L for the 32 isolated clones.

B: Summary of the different growth phenotypes of the isolated clones.


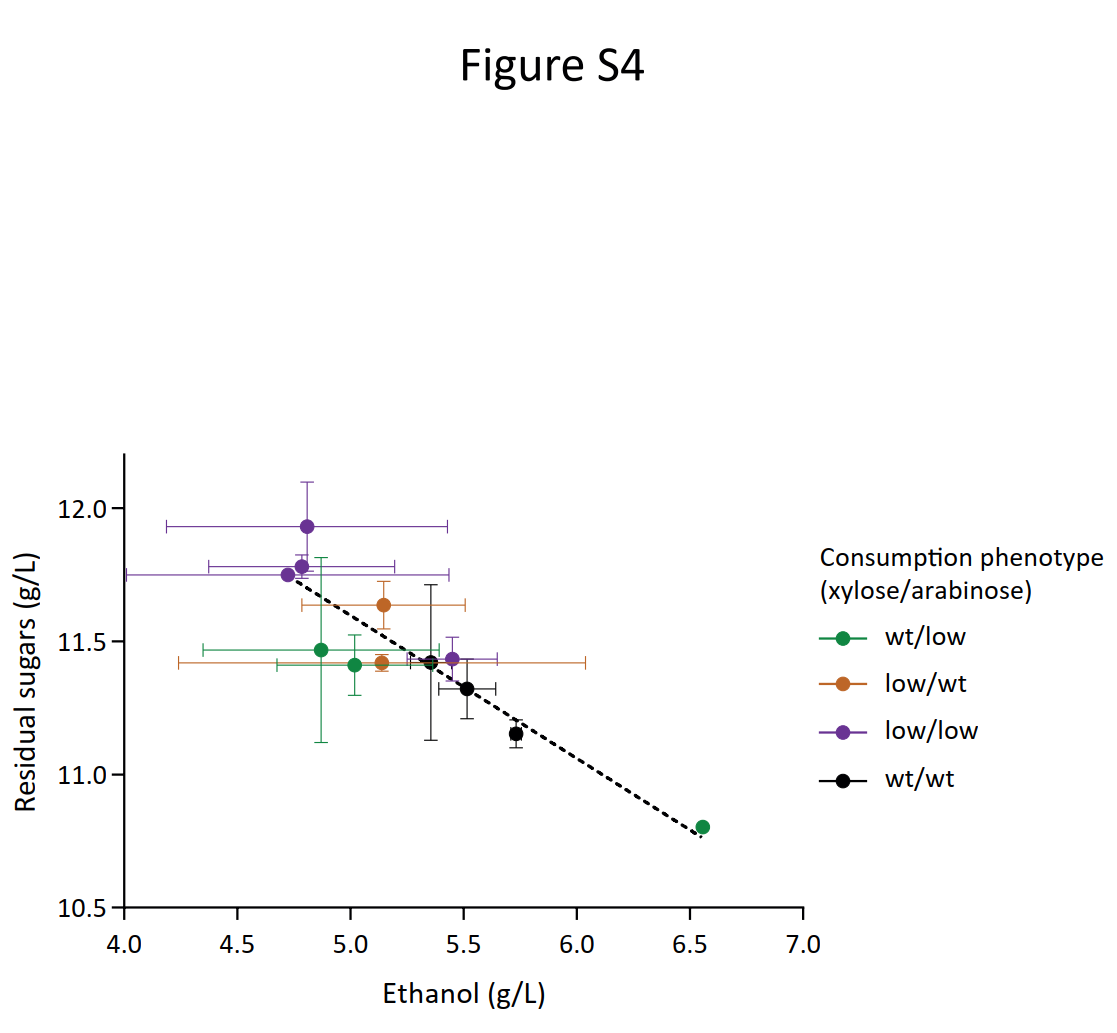


**Supplementary Figure 4.** **Correlation between C5 sugars consumption and ethanol production of selected clones isolated from batch cultures.**

Extracellular xylose, arabinose and ethanol were quantified after 24 h in YNB medium containing 16 g/L D-glucose, 13 g/L D-xylose and 11 g/L L-arabinose (n=2).


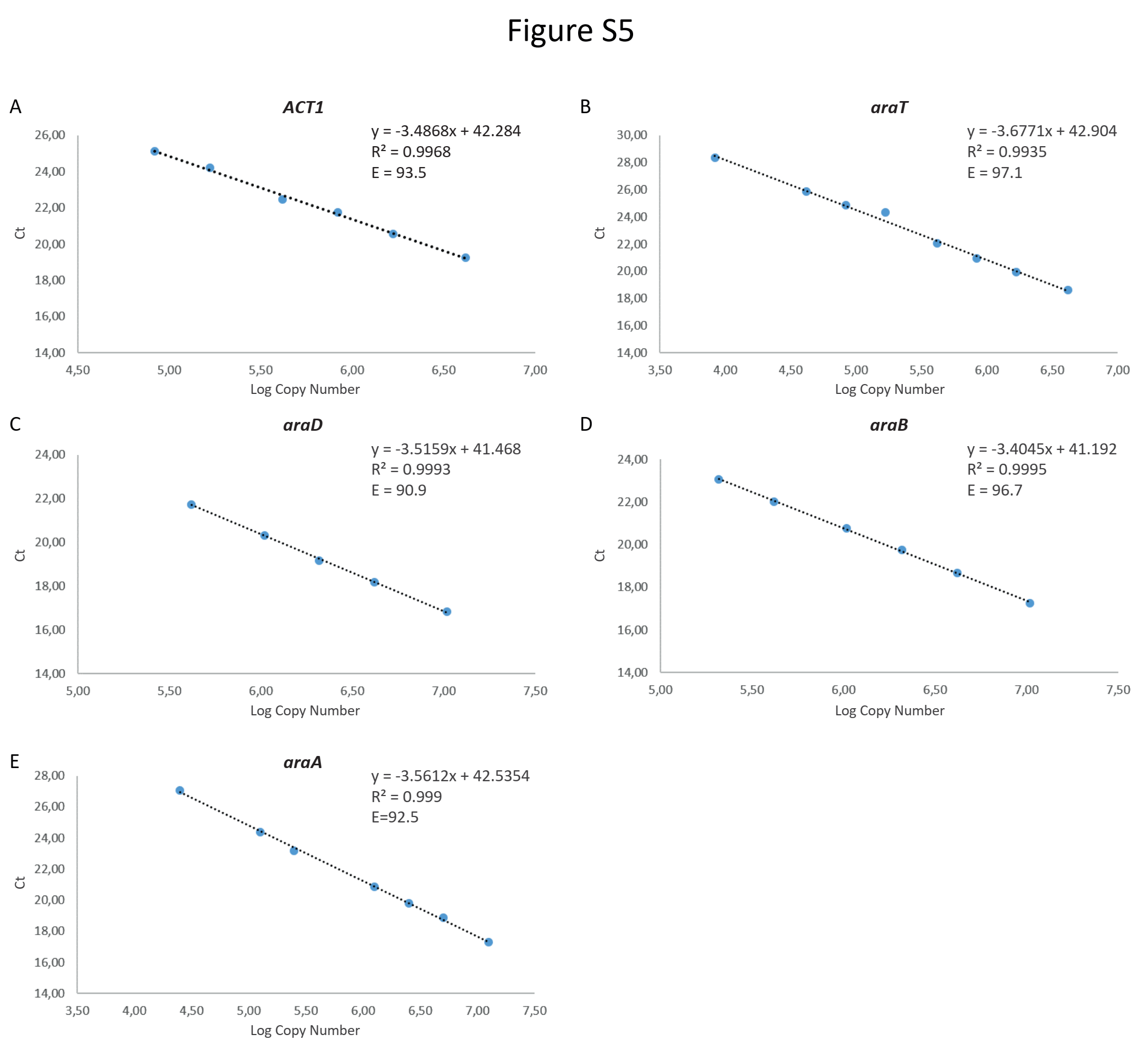


**Supplementary Figure 5.** **Standard curves used for qPCR assays calibration.**

Relationship between Cts and the logarithm of the copy number of *ACT1* (A), *araT* (B), *araD* (C), *araB* (D) and *araA* (E). Regression line parameters and percentage of efficiency (E%) based on each experiment are listed on curves.


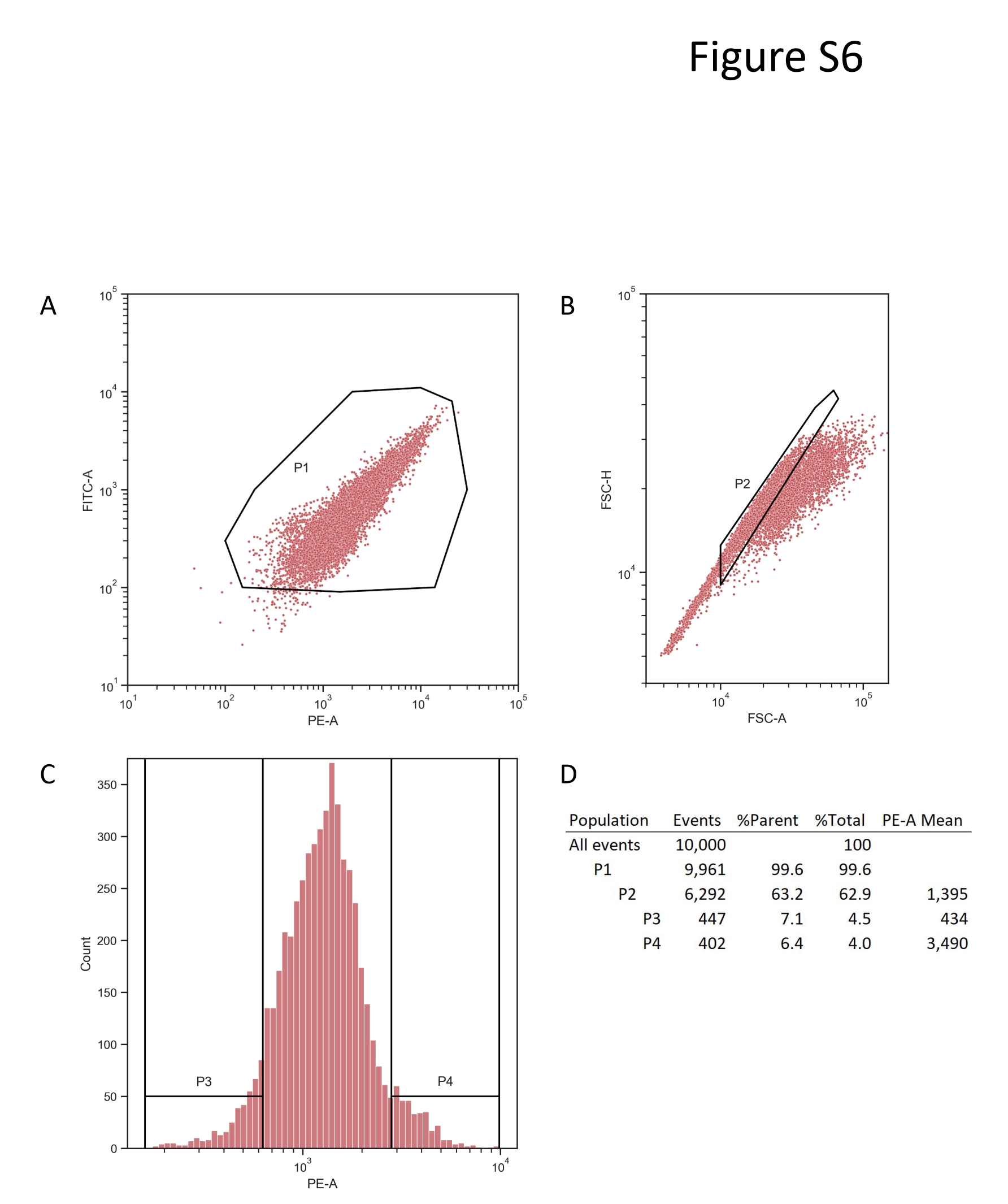


**Supplementary Figure 6.** **Gates applied during the cell sorting experiments.**

Data from the CO cultivation cycle are shown as an example. Ten thousand events were collected to define the gates applied for subsequent cell sorting.

A: The first gate (P1) was applied to select cells based on YFP (FITC-A) and tdTomato (PE-A) fluorescence signals.

B: The P2 gate was applied on P1-included cells to select single cells with similar cell size and granularity, based on the FSC-A vs. FSC-H plot.

C: Based on the histogram of the fluorescence, single cells with the lowest (P3) and the highest (P4) fluorescence levels were applied and used for cell sorting.

D: Cells number details found in each gate.


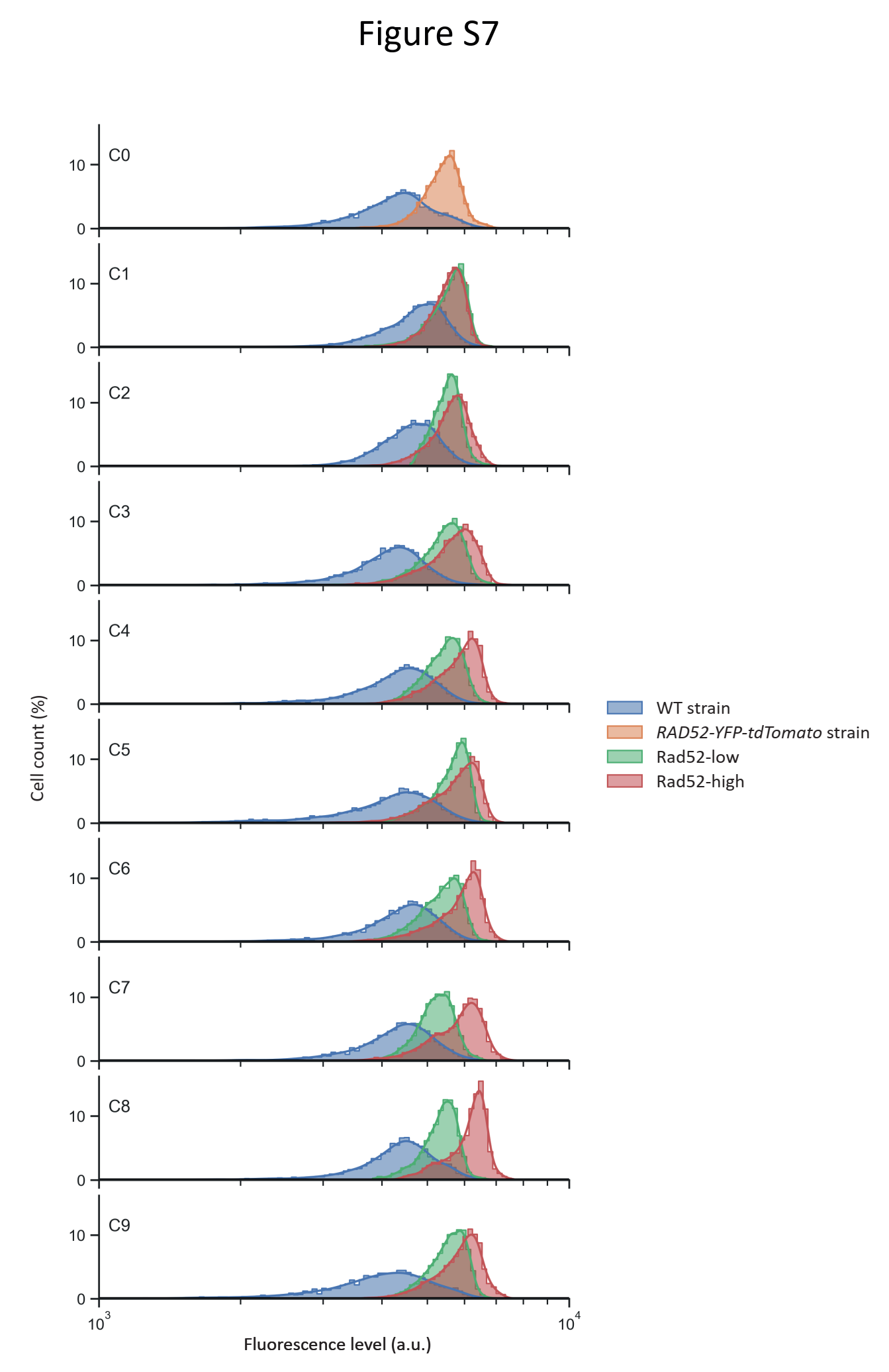


**Supplementary Figure 7.** **Evolution of the fluorescence profile of the strains during the successive cultivations in microplates.**

**Supplementary Table 1.** **Evolution of the biomass during the successive batch cultures.**

**Supplementary Table 2.** **Evolution of the biomass during successive cultivations in microplates.**

Initial OD was calculated based on the sorted cell number from the estimation that there are about 10^7^ cells/mL for an OD=1.

**Supplementary Table 3.** **List of primers used for qPCR assays.**

**Supplementary Table 4.** **Summary of the genomic variants identified in populations from successive cultivations in microplates.**

Genotype is indicated as 0 when the variant is absent and as 1 when the variant is present. In brackets is the number of reads getting the variant on the number total of reads at this position. The differences are bolded.
